# Early fever in patients with primary intracerebral hemorrhage is associated with worse long-term functional outcomes: a prospective study

**DOI:** 10.1186/s12883-023-03426-w

**Published:** 2023-10-19

**Authors:** Wen-Che Tseng, Yi-Hsiang Chiu, Yun-Chang Chen, Hsin-Shui Chen, Ming-Yen Hsiao

**Affiliations:** 1 Department of Physical Medicine and Rehabilitation, National Taiwan University Hospital, Yunlin Branch, 579, Sec. 2, Yunlin Rd, Douliu City, Yunlin County Taiwan; 2 Department of Physical Medicine and Rehabilitation, National Taiwan University Hospital, 7, Zhongshan S. Rd, Taipei, Taiwan; 3 Department of Physical Medicine and Rehabilitation, College of Medicine, National Taiwan University, 7, Zhongshan S. Rd, Taipei, Taiwan

**Keywords:** Intracerebral hemorrhage˙functional outcome˙prognosis˙survivors ˙ fever

## Abstract

**Background:**

Primary intracerebral hemorrhage (ICH) accounts for 85% of ICH cases and is associated with high morbidity and mortality rates. Fever can cause secondary injury after ICH; however, relevant studies have reported inconsistent results regarding the effects of fever on functional outcomes after ICH. This study examined the effects of early fever on the prognosis of ICH, particularly on long-term functional outcomes.

**Methods:**

This prospective study recruited patients with primary ICH at a tertiary medical center between 2019 and 2021. Early fever was defined as a tympanic body temperature of ≥ 38 °C upon admission. Barthel Index (BI) and modified Rankin scale (mRS) were examined at 1 year after ICH. A BI of ≤ 60 or mRS of ≥ 4 was considered as indicating severe disability.

**Results:**

We included 100 patients, and early fever was significantly associated with less functional independence at 1 year post-ICH, as determined using the mRS (p = 0.048; odds ratio [OR] = 0.23), and with severe functional dependency at 1 year post-ICH, as determined using the BI (p = 0.043; OR = 3) and mRS (p = 0.045; OR = 3). In addition, patients with early fever had a longer length of hospital stay (p = 0.002; 95% confidence interval = 21.80–95.91).

**Conclusions:**

Fever is common among patients with primary ICH. Our data indicate a significant association between early fever and worse functional outcomes in ICH survivors at 1 year after ICH. Additionally, patients with early fever had a significantly longer length of hospital stay after ICH.

**Supplementary Information:**

The online version contains supplementary material available at 10.1186/s12883-023-03426-w.

## Background

Nontraumatic intracerebral hemorrhage (ICH) constitutes10–15% of all stroke cases and is associated with high morbidity and mortality. Primary ICH accounts for 85% of all ICH cases and is associated with high morbidity and mortality rates [1]. ICH can lead to primary or secondary brain injury. Primary brain injury involves initial damage to the parenchyma induced by the blood clot, and secondary brain injury involves damage to blood vessels caused by various mechanisms, including oxidative stress, excitotoxicity, inflammatory response, mitochondrial dysfunction, and cell death [1, 2]. Excessive production of reactive oxygen species (ROS) leads to excessive oxidative stress, which aggravates secondary brain injury [2]. The aforementioned processes may be accelerated by fever. Therefore, fever is considered a potential contributor to secondary brain injury after ICH [3]. Previous study showed that hyperthermia was associated with poor outcomes at 3 months in both ischemic and hemorrhagic stroke patients, though the underlying mechanism may be different [4]. Fever is common in patients with ICH and is not usually associated with a clear infectious etiology. A study showed around 40% spontaneous ICH patients developed fever, but only 9% had an infectious etiology [5]. Studies have reported that patients with ICH with early fever had higher short-term mortality rates than did other patients; nevertheless, such studies have reported conflicting results regarding the effects of fever on functional outcomes after ICH [3, 6, 7]. In addition, most studies have applied a retrospective design and have used only the modified Rankin scale (mRS) score as their outcome measure. Extant studies have also used short follow-up periods; consequently, the long-term effects of fever in ICH survivors remain unclear [3]. To address these limitations, we conducted this prospective study to examine the effects of early fever on the prognosis of primary ICH; in particular, we examined the effects of fever on long-term functional outcomes in patients with primary ICH.

## Methods

### Study population

This prospective observational study examined 1-year functional outcomes in patients with primary ICH undergoing poststroke rehabilitation between 2019 and 2021 at a tertiary medical center. Patients were included in this study if they [1] were aged ≥ 20 years; [2] were admitted to a neurology, neurosurgery, or rehabilitation ward with a primary diagnosis of primary ICH, which was confirmed using a brain computed tomography or magnetic resonance imaging (MRI) scan; and [3] had provided written informed consent themselves or through their legal representatives. Patients were excluded if they died before discharge or transferred from other hospital for rehabilitation (i.e. not in early stage of ICH). Follow-up assessments were performed at 3, 6, and 12 months after the onset of ICH; the assessments involved phone interviews conducted by a physician.

### Outcome measures

Early fever was defined as a tympanic body temperature of ≥ 38 °C upon admission. The duration from the symptom onset to admission was recorded. All patients with fever upon admission were treated with acetaminophen as anti-pyretic treatment. mRS scores and Barthel Index (BI) values at 3, 6, and 12 months were the primary outcome measures (Appendix A, B). An mRS score of ≥ 4 was considered as indicating severe disability, and a BI value of ≤ 60 was considered as indicating severe dependency in activities of daily living (ADLs). Moreover, an mRS score of ≤ 2 or a BI value of > 90 was considered as indicating functional independence. The length of hospital stay was recorded by physicians through chart reviews and phone interviews (Appendix C).

### Statistical analysis

All statistical analyses were conducted using Stata software (version 14.0; StataCorp LLC, USA). Patient demographics and baseline characteristics were analyzed using descriptive statistics and are presented herein as mean ± standard deviation or percentage. Pearson’s chi-square test was used to examine the association between categorical variables, and a t test was used to examine the association between numerical variables and early fever. Multiple linear and logistic regression analyses were conducted to assess the association between early fever and functional outcomes. Area under the receiver operating characteristic curve (AUC) curve was also analyzed. P < 0.05 was considered statistically significant.

## Results

### Baseline characteristics

**Fig. 1:**
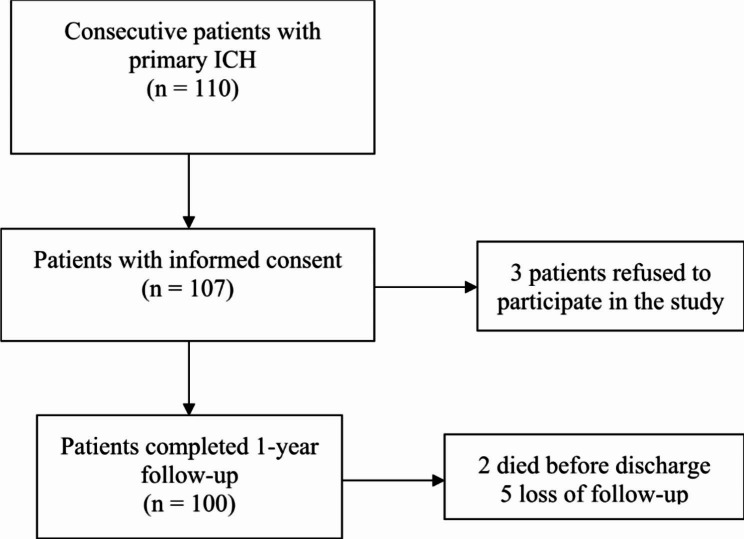
The study population

We collected the data of 100 consecutive patients (mean age: 60.69 years) who completed 1-year follow-up (Fig. 1). Of the included patients, 60% were men. The mean ICH volume was 20.34 mL. The mean initial National Institute of Health Stroke Scale (NIHSS) score was 14.16 (median: 14). The mean BI at admission was 15.24, and 16% of the patients had early fever upon admission. 97% patients had admission within 24 h from symptom onset, and the median time from onset to admission was 1 h. Among the patients with early fever, 4 had infectious fever and 12 had non-infectious fever upon admission. 3 patients were diagnosed as having pneumonia, and 1 patient had urinary tract infection. All the 4 patients with infectious fever were treated with antibiotics (3 with Ampicillin-Sulbactam and 1 with Ceftriaxone) (Table 1).

**Table 1:** Baseline characteristics of the population (n = 100)

Variables	*N (% or SD)*
Age (years)	60.69 (13.11)
Gender	
Female	40 (40)
Male	60 (60)
ICH volume (mL)	20.34 (21.83)
Hypertension	85 (85)
Diabetes mellitus	24 (24)
Dyslipidemia	30 (30)
Initial NIHSS	14.16 (7.29)
ICH score	
0	40 (43.01)
1	26 (27.96)
2	19 (20.43)
3	6 (6.45)
4	2 (2.15)
Infratentorial ICH origin	12 (12)
Ventriculostomy	7 (7)
Surgical hematoma evacuation	22 (22)
Presence of fever at admission	16 (16)
Infectious fever	4 (4)
Non-infectious fever	12 (12)
History of smoking	20 (20)
Mean BI at admission	15.24 (18.85)

N, number; SD, standard deviation; ICH, intracerebral hemorrhage; NIHSS, National Institute of Health Stroke Scale; BI, Barthel Index

### Functional outcomes at 1 year

The mean BI improved from 15.24 at admission to 72.22 at 1 year. Furthermore, at the 1-year follow-up, 29 patients (29%) exhibited severe dependency in ADLs. By contrast, at the 1-year follow-up, 41 (41%) patients achieved functional independence, as determined on the basis of a BI value of > 90; additionally, 34 (34%) patients achieved functional independence, as determined on the basis of an mRS score of $\le$2 (Tables 1 and 2). The location of hematoma was not significantly associated functional independence (p = 0.10, 95% confidence interval [CI] -1.17–0.10). Neither was surgery (p = 0.085, 95% CI -2.21–0.14), ventriculostomy (p = 0.28, 95% CI -3.35–0.97), or hematoma volume (p = 0.093, 95% CI -0.05–0.01). For functional dependence, logistic regression revealed nonsignificant results in location of hematoma (p = 0.28, 95% CI -0.21–0.073) and hematoma volume (p = 0.25, 95% CI -0.01–0.03), while surgery (p = 0.04, 95% CI -0.34–1.97) and ventriculostomy (p = 0.03, 95% CI 0.18–4.50) were associated with functional dependence. However, the association of surgery and ventriculostomy with functional dependence turned nonsignificant after adjusted for age and sex (p = 0.051, 95% CI -0.01–1.98 for surgery and p = 0.056, 95% CI 0.06–4.42 for ventriculostomy, respectively).

**Table 2:** Functional outcomes at one year (n = 100)

Variables	*N (% or SD)*
mRS	
1	13 (13)
2	21 (21)
3	26 (26)
4	23 (23)
5	9 (9)
6	2 (2)
Mean BI	72.22 (29.08)
Severe dependency	
BI$\le$60	29/100 (29)
mRS $\ge$4	40/100 (40)
Functional independence	
BI > 90	41/100 (41)
mRS $\le$2	34/100 (34)

N, number; SD, standard deviation; BI, Barthel Index; mRS, modified Rankin Scale

### Early fever and functional outcomes

Our results revealed no significant difference in age, sex, initial Glasgow Coma Scale (GCS) score, or initial BI between patients with or without early fever (Table 3). At the 1-year follow-up, early fever was significantly associated with both lower chance of achieving functional independence defined as mRS score $\le$2 (p = 0.048; odds ratio [OR] = 0.23) and severe dependency in ADLs, defined as BI ≤ 60 (p = 0.043; OR = 3) and mRS score ≥ 4 (p = 0.045; OR = 3) (Table 3).

**Table 3:** Comparison between ICH survivors with or without early fever (n = 100)

Variables	Early fever (n = 16) *N (% or SD)*	Without early fever (n = 84) *N (% or SD)*	*P*-value	*Odds Ratio* *(OR)*
Age (years)	56.63.26 (11.67)	61.46 (13.30)	0.15	
Gender (female)	7/16 (43.75)	33/84 (39.29)	0.74	
Initial GCS	12.69 (3.73)	12.58 (3.21)	0.91	
Initial BI	13.75 (27.5)	15.31 (18.26)	0.87	
Functional independence at 1-year				
BI > 90	4/16 (25)	37/84 (44.05)	0.16	0.32^†^
mRS $\le$2	2/16 (12.5)	32/84 (38.1)	0.048*	0.23^†^
Severe dependency at 1-year				
BI$\le$60	8/16 (50)	21/84 (25)	0.043*	3^‡^
mRS $\ge$4	10/16 (62.5)	30/84 (35.72)	0.045*	3^‡^
Surgery	7/16 (43.75)	15/84 (17.86)	0.02*	3.58
Ventriculostomy	2/16 (12.5)	5/84 (5.96)	0.35	1.98
Length of hospital stay (days)	123.62 (100.48)	66.24 (52.29)	0.002*	

*p < 0.05

† Odds ratio for better outcome (BI > 90 or mRS score$\le$2)

‡ Odds ratio for worse outcome (BI ≤ 60 or mRS score ≥ 4)

N, number; SD, standard deviation; GCS, Glasgow coma scale; BI, Barthel Index; mRS, modified Rankin Scale

After adjusted for age and sex, early fever was not significantly associated with functional independence (defined as mRS score$\le$2) at 3 months (p = 0.18, 95% CI -3.62–0.66) or 6 months (p = 0.08, 95% CI -3.01–0.17) (data not shown in the table), but the association turned significant at 1-year (p = 0.031, 95% CI -3.39–0.17) (Table 4). For functional dependence (defined as mRS score of ≥ 4), logistic regression revealed nonsignificant results at 3 months (p = 0.061, 95% CI -0.06–2.42), but the association turned significant at both 6 months (p = 0.01, 95% CI 0.52–3.10) (data not shown in the table) and 1-year (p = 0.025, 95% CI 0.17–2.52) (Table 4).

**Table 4:** Early fever and functional outcomes at one year after ICH (n = 100)

Model	p-value	95% CI	AUC
**Functional independence**			
BI > 90 ^†^	0.085	(-2.37, 0.15)	0.6714
mRS $\le$2 ^†^	0.031*	(-3.39, -0.17)	0.7143
**Functional dependence**			
BI$\le$60 ^†^	0.003*	(0.34, 1.73)	0.7088
mRS$\ge$4 ^†^	0.025*	(0.17, 2.52)	0.6848
**Hospital Stay** ^‡^	0.002*	(21.80, 95.91)	NA

BI, Barthel Index; mRS, modified Rankin Scale; ICH, intracerebral hemorrhage; CI, confidence interval; AUC, area under the receiver operating characteristic curve; NA, not applicable

*p < 0.05

† Logistic regression, adjusted for age and sex

‡ Multiple linear regression, adjusted for age and sex

In addition, early fever was associated with a longer length of hospital stay after ICH (p = 0.002; 95% CI = 21.80–95.91 days) and was associated with potential surgery during the treatment course (p = 0.02; OR = 3.58; Table 3).

### Different fever definitions and functional outcomes

The authors performed further analysis examining functional outcomes at 1 year by different definitions of fever. If fever was defined as body temperature$\ge$ 37.5 °C, early fever was significantly associated with functional independence (defined as mRS score$\le$2) (p = 0.023, 95% CI -2.65–0.19) but not functional dependence (defined as mRS score of ≥ 4) at 1 year (p = 0.066, 95% CI -0.06–1.91).

If fever was defined as body temperature$\ge$37°C, early fever was not significantly associated with functional independence (p = 0.082, 95% CI -0.97–0.77) nor functional dependence (p = 0.38, 95% CI -1.23–0.46) at 1 year.

## Discussion

### Early fever and functional outcomes after ICH

The present study is the first to examine the association between early fever and long-term functional outcomes in ICH survivors. Our findings reveal that early fever at admission was associated with worse functional outcomes at 1 year after ICH in patients undergoing rehabilitation and was associated with a longer length of hospital stay after ICH. By contrast, a 2022 review of 19 clinical studies concluded that fever was associated with an increased risk of short-term mortality but was not associated with poor outcomes among ICH survivors [3]. This discrepancy may stem from differences in sample characteristics, fever definitions, and temperature measurements. In addition, most studies were retrospective, and some included patients with a diagnosis other than primary ICH. The largest study involved post hoc analyses of the INTERACT2 trial, which randomly assigned 2839 patients to intensive (< 140 mmHg) or guideline-recommended (< 180 mmHg) blood pressure (BP) management after acute cerebral hemorrhage [8]. The post hoc analyses showed that early pyrexia (≥ 37.5 °C) upon admission is associated with a larger perihematomal edema volume in 24 h and a higher mortality risk but not with major disability (mRS ≥ 3) at 3 months [9]. However, the mentioned study included only patients with mild to moderate ICH with elevated BP (> 150 mmHg), and the follow-up duration was relatively short. Another prospective study with a longer follow-up duration demonstrated that fever is associated with worse health-related quality of life at 1 year; nevertheless, the mentioned study did not assess other measures of functional outcomes [10]. As for the etiology of early fever in ICH patient, it could be dichotomized as infectious or non-infectious (central) fever [7]. One-fourth of the patients with early fever in the present study were diagnosed as having infectious fever, and the ratio of infectious to non-infectious fever was similar to previous studies [6, 7]. Studies for primary ICH patients showed that both infectious and non-infectious fever were associated with worse outcomes in short term (at discharge or at 3 months) [6, 7]. However, a subgroup analysis in the present study was not performed due to the small sample size.

### Explanation of worse outcomes associated with fever after ICH

In ischemic stroke, fever aggravates secondary brain injury through increases in inflammatory response, blood–brain barrier permeability, leukocyte recruitment and activation, and free-radical production [11, 12]. By contrast, the mechanism through which fever contributes to secondary brain injury in ICH remains poorly understood. Two preclinical studies have reported that hematoma extension and functional outcomes after ICH in young adult male rats were not influenced by hyperthermia in the short term [13, 14]. However, in animal models, hyperthermia is induced through physical heating methods, such as an infrared lamp; these methods may differ from the means through which fever and the subsequent cascade are induced in patients with ICH. The post hoc analyses of the INTERACT2 trial revealed that early hyperthermia is associated with worse cerebral edema in patients with ICH, [9] implying that the mechanism underlying poor outcomes after ICH may be comparable to that in ischemic stroke. However, additional studies are warranted to more comprehensively investigate the mechanism through which hyperthermia worsens outcomes after ICH. In the present study, the difference in functional outcomes between patients with or without early fever turned significant gradually with time (as shown in Results Sect. 3.3), which implied that early fever could potentially impact the outcomes of ICH by delaying recovery rather than exacerbating the initial injury. If this hypothesis is true, studies examining early fever and ICH outcomes may not be able to detect the difference in prognosis between two groups without a long-term follow-up. This may help explain why previous studies on ICH showed inconsistent results.

The investigators also examined the association between early fever and functional outcomes by various fever definitions as sensitivity analyses (Results Sect. 3.4), and the theory that hyperthermia worsens outcomes after ICH was further consolidated by the results.

Therapeutic hypothermia is an emerging intervention for ICH. Two systematic reviews and meta-analyses including ≥ 18 preclinical and clinical studies have demonstrated that therapeutic hypothermia yields significant benefits in behavioral outcomes and cerebral edema. However, heterogeneity between studies in terms of cooling protocols and reported clinical outcomes precluded a definitive conclusion [15, 16]. Although the exact mechanism underlying the effects of therapeutic hypothermia remains unclear, it may reduce free radicals and endothelial cell swelling and prevent inflammatory cells from entering the injured brain [17].

### Early fever and length of hospital stay

Few studies have evaluated the effect of fever on the length of hospital stay in patients with ICH. We observed that ICH survivors with early fever had a prolonged length of hospital stay (p = 0.002; 95% CI = 21.80–95.91). By contrast, a 2017 retrospective study including 351 patients with ICH observed that ICH survivors with fever had a shorter length of hospital stay than did patients without fever [7]. Taiwan’s National Health Insurance system covers in-patient rehabilitation after stroke for up to 6 months and may thus alleviate any bias in the results due to heterogeneity in health-care access.

### Limitations

This study has several limitations. First, this was a single-center study with a relatively small sample size. Second, because this was not an interventional study, no standard protocol was followed for fever management, possibly leading to considerable interindividual variations in terms of treatment effects. Third, the follow-up was conducted through phone interviews rather than on-site interviews, which may have resulted in bias. Fourth, the present study excluded patients who died before discharge, which impair the generalizability to all ICH patients. Finally, body temperature was measured using the tympanic method. Therefore, our findings may differ from those of other studies using other methods of measuring body temperature, such as axillary or rectal temperature measurement methods.

## Conclusions

This prospective study revealed a significant association between early fever and worse functional outcomes at 1 year after ICH. In addition, patients with ICH presenting with fever at admission (early fever) had a significantly prolonged length of hospital stay.

### Electronic supplementary material

Below is the link to the electronic supplementary material.

Supplementary Material 1

## Data Availability

The data associated with the paper are not publicly available but are available from the corresponding author on reasonable request.

## Abbreviations

ICH: Intracerebral hemorrhage
ROS: Reactive oxygen species
mRS: Modified Rankin Scale
CT: Computed tomography
MRI: Magnetic resonance imaging
BI: Barthel Index
ADLs: Activities of daily living
GCS: Glasgow Coma Scale
IVH: Intraventricular hemorrhage
AUC: Area under the receiver operating characteristic curve
SD: Standard deviation
OR: Odds ratio
CI: Confidence interval

## References

[1] Rajashekar D, Liang JW. Intracerebral hemorrhage. StatPearls. Treasure Island (FL): StatPearls Publishing. Copyright. © 2022, StatPearls Publishing LLC.; 2022.

[2] Shao L, Chen S, Ma L (2022). Secondary Brain Injury by oxidative stress after cerebral hemorrhage: recent advances. Front Cell Neurosci.

[3] Liddle LJ, Dirks CA, Almekhlafi M, Colbourne F. An ambiguous role for fever in worsening Outcome after Intracerebral Hemorrhage. Transl Stroke Res. 2022. 10.1007/s12975-022-01010-x PMC9995537 35366212

[4] Campos F, Sobrino T, Vieites-Prado A, Pérez-Mato M, Rodríguez-Yáñez M, Blanco M (2013). Hyperthermia in human ischemic and hemorrhagic stroke: similar outcome, different mechanisms. PLoS ONE.

[5] Iglesias-Rey R, Rodríguez-Yáñez M, Arias S, Santamaría M, Rodríguez-Castro E, López-Dequidt I (2018). Inflammation, edema and poor outcome are associated with hyperthermia in hypertensive intracerebral hemorrhages. Eur J Neurol.

[6] Honig A, Michael S, Eliahou R, Leker RR (2015). Central fever in patients with spontaneous intracerebral hemorrhage: predicting factors and impact on outcome. BMC Neurol.

[7] Gillow SJ, Ouyang B, Lee VH, John S (2017). Factors Associated with Fever in Intracerebral Hemorrhage. J Stroke Cerebrovasc Dis.

[8] Anderson CS, Heeley E, Huang Y, Wang J, Stapf C, Delcourt C (2013). Rapid blood-pressure lowering in patients with acute intracerebral hemorrhage. N Engl J Med.

[9] Malavera A, You S, Zheng D, Delcourt C, Anderson CS (2021). Prognostic significance of early pyrexia in acute intracerebral haemorrhage: the INTERACT2 study. J Neurol Sci.

[10] Bush RA, Beaumont JL, Liotta EM, Maas MB, Naidech AM (2018). Fever burden and health-related quality of Life after Intracerebral Hemorrhage. Neurocrit Care.

[11] Castillo J, Dávalos A, Noya M (1999). Aggravation of acute ischemic stroke by hyperthermia is related to an excitotoxic mechanism. Cerebrovasc Dis.

[12] Polderman KH (2008). Induced hypothermia and fever control for prevention and treatment of neurological injuries. Lancet.

[13] Williamson MR, Colbourne F (2017). Evidence for decreased brain parenchymal volume after large intracerebral hemorrhages: a potential mechanism limiting intracranial pressure rises. Transl Stroke Res.

[14] Wilkinson CM, Kalisvaart ACJ, Kung TFC, Maisey DR, Klahr AC, Dickson CT (2020). The collagenase model of intracerebral hemorrhage in awake, freely moving animals: the effects of isoflurane. Brain Res.

[15] Baker TS, Durbin J, Troiani Z, Ascanio-Cortez L, Baron R, Costa A (2022). Therapeutic hypothermia for intracerebral hemorrhage: systematic review and meta-analysis of the experimental and clinical literature. Int J Stroke.

[16] Melmed KR, Lyden PD (2017). Meta-analysis of pre-clinical trials of therapeutic hypothermia for Intracerebral Hemorrhage. Ther Hypothermia Temp Manag.

[17] Wu TC, Grotta JC (2013). Hypothermia for acute ischaemic stroke. Lancet Neurol.

