# Supplementary material for: Early fever in patients with primary intracerebral hemorrhage is associated with worse long-term functional outcomes: a prospective study
Source: BMC Neurol. 2023 Oct 19;23:375. doi: 10.1186/s12883-023-03426-w (PMC10585771; doi:10.1186/s12883-023-03426-w)
Supplement: Supplementary file 1 — Supplementary Material 1 [file 12883_2023_3426_MOESM1_ESM.docx]

**Appendix**

1. **Barthel index**

| **Activity** | **Score** |
| --- | --- |
| Feeding | 0 = unable  5 = needs help cutting, spreading butter, etc., or requires modified diet  10 = independent |
| Bathing | 0 = dependent  5 = independent (or in shower) |
| Grooming | 0 = needs to help with personal care  5 = independent face/hair/teeth/shaving (implements provided) |
| Dressing | 0 = dependent  5 = needs help but can do about half unaided  10 = independent (including buttons, zips, laces, etc.) |
| Bowels | 0 = incontinent (or needs to be given enemas)  5 = occasional accident  10 = continent |
| Bladder | 0 = incontinent, or catheterized and unable to manage alone  5 = occasional accident  10 = continent |
| Toilet | 0 = dependent  5 = needs some help, but can do something alone  10 = independent (on and off, dressing, wiping) |
| Transfer | 0 = unable, no sitting balance  5 = major help (one or two people, physical), can sit  10 = minor help (verbal or physical)  15 = independent |
| Ambulation | 0 = immobile or < 50 yards  5 = wheelchair independent, including corners, > 50 yards  10 = walks with help of one person (verbal or physical) > 50 yards  15 = independent (but may use any aid; for example, stick) > 50 yards |
| Stairs | 0 = unable  5 = needs help (verbal, physical, carrying aid)   1. independent |

1. **Modified Rankin scale**

| **Score** | **Definition** |
| --- | --- |
| 0 | No symptoms |
| 1 | No significant disability. Able to carry out all usual activities, despite some symptoms. |
| 2 | Slight disability. Able to look after own affairs without assistance, but unable to carry out all previous activities. |
| 3 | Moderate disability. Requires some help, but able to walk unassisted. |
| 4 | Moderately severe disability. Unable to attend to own bodily needs without assistance, and unable to walk unassisted. |
| 5 | Severe disability. Requires constant nursing care and attention, bedridden, incontinent. |
| 6 | Dead. |

1. **Phone interview form**

**Date of Interview:**

**Part I: Patient Information**

1. **Name:**
2. **Chart number:**
3. **Age:**
4. **Sex:**
5. **Onset of Stroke:**
6. **Diagnosis**
7. **Caregiver:**
8. **Current residence (e.g. home, nursing facility):**

**Part II: Functional status**

1. **Please choose the statement that most closely corresponds to your current level of ability to perform the following daily activities:**
   1. **Feeding**
      1. Unable to feed yourself
      2. Needs help cutting, spreading butter, etc., or requires modified diet
      3. Independent
   2. **Bathing**
      1. Dependent
      2. Independent (in and out of the bath or shower)
   3. **Grooming**
      1. Needs help with personal care
      2. Independent face/hair/teeth/washing, shaving (implements provided)
   4. **Dressing**
      1. Dependent
      2. Needs help but can do about half unaided
      3. Independent (including buttons, zips, laces, etc.)
   5. **Bowel Control**
      1. Incontinent or needs help for enemas
      2. Occasional accident
      3. Continent
   6. **Bladder Control**
      1. Incontinent, or catheterized and unable to manage by yourself
      2. Occasional accident
      3. Continent
   7. **Toilet Use**
      1. Dependent
      2. Needs help in transferring, cleaning, or managing clothes
      3. Independent
   8. **Transfers (bed to chair, wheelchair, etc.)**
      1. Unable
      2. Major help (one or two people, physical assistance), can sit
      3. Minor help (verbal or physical assistance)
      4. Independent
   9. **Mobility (on level surfaces)**
      1. Immobile or less than 50 meters
      2. Wheelchair independent, more than 50 meters
      3. Walks with help of one person (verbal or physical), more than 50 meters
      4. Independent (may use any aids), more than 50 meters
   10. **Stairs**
       1. Unable
       2. Needs help (verbal or physical)
       3. Independent
2. **Are you able to carry out all usual activities?**
3. **Do you have any indwelling tubes currently (includes but not limits to nasogastric tube and Foley catheter)?**

**Part III: Rehabilitation**

1. **Are you receiving any in-patient or out-patient rehabilitation program currently?**
2. **The duration of your in-patient and out-patient programs?**
3. **The types of rehabilitation received (e.g., physical therapy, occupational therapy, speech therapy)?**
4. **Is there anything else you would like to add or any other experiences you would like to share?**

Note: A proxy interview will be performed if the patient is unable to complete the interview (e.g. aphasia, dementia, impaired consciousness)
